# Ancient DNA from Coral-Hosted *Symbiodinium* Reveal a Static Mutualism over the Last 172 Years

**DOI:** 10.1371/journal.pone.0055057

**Published:** 2013-02-06

**Authors:** David M. Baker, Lee Weigt, Marilyn Fogel, Nancy Knowlton

**Affiliations:** 1 Smithsonian Institution, Marine Science Network, Washington, D.C., United States of America; 2 Geophysical Laboratory, Carnegie Institution of Washington, Washington, D.C., United States of America; 3 Smithsonian Institution, Laboratories of Analytical Biology, Museum Support Center, Suitland, Maryland, United States of America; 4 Smithsonian Institution, Department of Invertebrate Zoology, Washington, D.C., United States of America; University of Canterbury, New Zealand

## Abstract

Ancient DNA (aDNA) provides powerful evidence for detecting the genetic basis for adaptation to environmental change in many taxa. Among the greatest of changes in our biosphere within the last century is rapid anthropogenic ocean warming. This phenomenon threatens corals with extinction, evidenced by the increasing observation of widespread mortality following mass bleaching events. There is some evidence and conjecture that coral-dinoflagellate symbioses change partnerships in response to changing external conditions over ecological and evolutionary timescales. Until now, we have been unable to ascertain the genetic identity of *Symbiodinium* hosted by corals prior to the rapid global change of the last century. Here, we show that *Symbiodinium* cells recovered from dry, century old specimens of 6 host species of octocorals contain sufficient DNA for amplification of the ITS2 subregion of the nuclear ribosomal DNA, commonly used for genotyping within this genus. Through comparisons with modern specimens sampled from similar locales we show that symbiotic associations among several species have been static over the last century, thereby suggesting that adaptive shifts to novel symbiont types is not common among these gorgonians, and perhaps, symbiotic corals in general.

## Introduction

Many diverse cnidarians, including scleractinian corals and their alcyonarian and gorgonian soft coral relatives have evolved obligate, intra-cellular symbioses with dinoflagellate algae of the genus *Symbiodinium*. These algae are themselves diverse, representing at least 9 divergent lineages or “clades” [1], [2]. Some of these clades contain considerable diversity (‘types’), suggesting that species level boundaries may exist at sub-clade resolutions [3]. Currently, our understanding of the functional significance of symbiont diversity is based on zonation patterns of symbiont clades within and among corals distributed across the reefscape [4], [5], and carbon-centered metrics of photosynthetic performance under varied conditions [6], [7]. These physiological differences may be linked to a range of tolerances to environmental stressors including sedimentation, high irradiance, and most importantly, extreme temperatures among corals hosting distinct *Symbiodinium* clades [8]–[11]. Global observations of thermal tolerance and maintenance of symbiosis (bleaching resistance) exhibited by various clades and sub-clade types have led to the hypothesis that the process of bleaching may itself be an adaptive mechanism for acquiring novel, stress-tolerant symbionts, which could serve to increase the host’s survival during bouts of environmental change [12]–[13]. To test this hypothesis is to ask; have symbiont types changed since the onset of anthropogenic climate warming over the last century?

One way of answering this question is by identifying *Symbiodinium* from corals sampled prior to major anthropogenic global change. Sources of such materials can be found in museum archives [14]. Unfortunately, as scleractinian morphological taxonomy is based on skeletal structures, most museum held hard coral specimens collected over the last 200 years have been bleached to remove all organic tissues, while wet collections of hard corals are less common. Alternatively, gorgonian corals are often preserved dry with the outer tissues (coenenchyme), including the polyps and their *Symbiodinium* intact. The Smithsonian’s National Museum of Natural History contains over 6,000 specimens of gorgonian corals, with a large representation of dried specimens. Remarkably, the collection is not limited to individual type specimens and contains many lots of independent colonies contemporaneously collected from the same geographic areas thus providing important replication for genetic analyses.

Gorgonians are globally distributed but the majority of species hosting *Symbiodinium spp.* are found on shallow reefs in the Caribbean Sea. These octocorals exhibit different colony morphologies, (primarily rod, fan, and plume) as well as variation in polyp size which is hypothesized to be related to prey-capture and therefore, reflective of the relative reliance on auto- vs. heterotrophic nutrition [15]. Thus, gorgonian species may differ with respect to their reliance on autotrophic nutrition translocated from their symbionts. Moreover, current observations on the flexibility of gorgonian-algal symbioses suggests that they are more constrained than scleractinians ([16]; but see also [17]). Nearly 90% of Caribbean gorgonians are primarily found in symbiosis with clade B *Symbiodinium* [18], though there are some examples of clade flexibility. For instance, *Plexaura homomalla* and *Eunicea tourneforti* have been observed hosting multiple types from clade C as well as clade B *Symbiodinium* [14]. Furthermore, experimental infections of newly settled juveniles of *Plexaura kuna* and *Pseudoplexaura porosa* demonstrated flexibility in host associations with clades A, B, and C [19]. Like scleractinians, the diversity of gorgonian-symbiont associations may explain patterns of holobiont tolerance to environmental stressors.

Published studies on the bleaching response to thermal stress among gorgonians have shown that some species are highly resistant [20]–[22] while others are susceptible [23] and can subsequently succumb to mortality [24]–[26]. Following the mass bleaching of 2005 in Puerto Rico, Prada (2010) observed widespread bleaching (observed whitening) among many genera, including *Muricea, Muriceopsis*, *Pseudoplexaura*, *Briarium, Pterogorgia* and *Plexaurella*, while *Gorgonia, Eunicea* (with the exception of *E. flexuosa*) and *Pseudopterogorgia* did not visibly bleach. However, visible signs of bleaching may only manifest after substantial loss of symbiont cells has already occurred [27] and host-derived pigments, particularly carotenoids associated with gorgonian sclerites may confound visual assessments of bleaching in gorgonian corals [28]. Nevertheless we can conclude from these limited observations that many gorgonians bleach and there is substantial variation in bleaching susceptibility among various genera. Given that living gorgonians have displayed instances of flexible symbioses with clade B and C sub-types [29], and the observation that bleached gorgonians can acquire novel symbionts from the water column [30] there is a basis for questioning whether anthropogenic change over the last century is associated with a shift in the predominant symbiont clades hosted by modern gorgonian corals.

Whether or not the process of bleaching is an adaptive mechanism, it seems clear that *Symbiodinium* diversity is tied to a spectrum of environmental tolerances. Therefore, shifts to tolerant types via natural selection over time would be indicative of adaptation to environmental stress. Given the near +1.0°C warming of the surface ocean over the last 150 years has pushed corals to the limits of their thermal tolerance and increased the occurrence of mass bleaching events [31] the main goal of this study was to determine if shifts in dominant symbiont types hosted by several gorgonian species has occurred. We also tested the null hypothesis that past populations of gorgonians separated by distance host the same clades, and that all host species associated with the same symbiont types.

## Methods

### Sample Collection and Preparation

Historical gorgonian specimens (*n = *82) were obtained from the dry collection of the Smithsonian National Museum of Natural History (NMNH; Table 1). Most of these specimens were small (<50 cm) and we estimate that these colonies were, on average, <20 years old at the age of collection based on size and band counts of basal cross-sections obtained from select specimens (data not shown). Thus, we estimate that the specimens sampled in this study likely represent up to 9 generations. We selected from the most abundant species in the NMNH collection, including the sea fans *Gorgonia ventalina* and *G. flabellum,* the sea plume *Pseudopterogorgia acerosa,* and the sea rod *Eunicea flexuosa.* These species have been found in association with several sub-clade types. *Gorgonia* and *Pseudopterogorgia* have been found in symbiosis with B1, and *Eunicea flexuosa* with B1, B1b, B2, and B8 [32].

**Table 1:** Summary of specimens.

Catalog Number	Scientific Name	n (seq.)	Collector(s)	Year Collected	Country	Precise Locality
59474	*Eunicea succinea*	1 (1)	J.E. Benedict	1901	USA	Caesar Creek, Florida
14388	*Eunicea flexuosa*	15 (6)	W. Nye	1886	Bahamas	New Providence Island
no ID		11 (8)	W.L. Schmitt	∼1905	USA	Dry Tortugas, Florida
no ID		5 (4)	unknown	1925	USA	Dry Tortugas, Florida
na		10 (5)	E. Bartels	2007	USA	Summerland Key, Florida
14766	*Gorgonia flabellum*	2 (1)	W. Nye	1886	Bahamas	Abaco Island
54232		12 (8)	P. Bartsch	1912	Bahamas	Andros Island
14400		1 (1)	no data	1886	Bahamas	Watlings Island
14397	*Gorgonia ventalina*	4 (3)	W. Nye	1886	Bahamas	New Providence Island
54232		6 (2)	P. Bartsch	1912	Bahamas	Andros Island
14400		2 (0)	unknown	1886	Bahamas	Watlings Island
na		5 (3)	D. Baker	2010	Bahamas	Lee Stocking Island
34779		3 (2)	Henderon and Barson	1914	Cuba	Santa Lucia Bay
8860		2 (2)	E. Palmer	1884	USA	Florida
8884		4 (1)	E. Palmer	1884	USA	Florida
95428		2 (0)	E. Palmer	1884	USA	Key West, Florida
33627		1 (1)	P. Bartsch*	1912*	USA	Biscayne Bay, Florida
1625		2 (2)	C. Pickering	1838–1842**	USA	Florida
54232		3 (1)	P. Bartsch	1912	USA	Biscayne Bay, Florida
na		7 (1)	E. Bartels	2007	USA	Summerland Key, Florida
8862	*Pseudopterogorgia acerosa*	6 (3)	E. Palmer	1884	USA	Carysfort Reef, Florida
8866		1 (0)	E. Palmer	1884	USA	Salt Pond Key, Florida
33614		11 (8)	J.E. Benedict	1901	USA	Carysfort Reef, Florida
6913	*Pterogorgia anceps*	1 (1)	H. Hemphill	1884	USA	Tampa Bay, Florida

n = total number of specimens sampled for this study.

(seq.) = total number of specimens yielding consensus sequences used in Fig. 1.

** Estimated year: Charles Pickering was a crew member of the United States Exploring Expedition at this time.

* Year estimated by collector/catalog number.

All museum specimens were collected from the Florida Keys and the Bahamas, though 3 specimens from Cuba were added to the Florida sample set. Each specimen or lot was accessed from storage cabinets organized by geographic origin and time of collection. Most specimens were enclosed in heavy plastic bags. For comparison, modern specimens of *G. ventalina* (*n = *7) and *E. flexuosa* (*n* = 10) were collected offshore of Summerland Key, FL in 2007 and *G. ventalina* (*n = *5) was collected from Lee Stocking Island, Bahamas in 2010 (Table 1). All modern samples were oven dried at 60°C to constant weight, ground into a powder, and stored in individual sealed tubes.

The storage and subsequent handling of both modern and museum specimens was conducted at separate locations throughout the duration of this study to eliminate the risk of cross-contamination between modern and historical specimens. Tissue grinding, DNA extraction, PCR, and cleanup were conducted in separate laboratories at different times using independent equipment and reagent kits up to the point of sequencing [33]. First, museum specimen work was conducted at the Laboratories of Analytical Biology at the Smithsonian’s Museum Support Center. Subsequently, all modern specimens were processed at the Geophysical Laboratory of the Carnegie Institution of Washington. Neither facility had conducted work on *Symbiodinium* DNA prior to this study. Approximately 3 g of tissue was removed by hand or using scissors and then homogenized using a mortar and pestle. To reduce cross contamination between samples, all equipment was washed with soap, rinsed with tap water, soaked in a bleach solution, and rinsed several times with deionized water followed by a final ethanol rinse to enhance drying. In between sets of samples from different species each mortar and pestle was autoclaved following the washing protocol.

Approximately 0.5–1 mL (vol.) of homogenized sample was placed in a 2.0 mL microcentrifuge tube, rehydrated with 1 mL of 0.2 µM sterile-filtered 0.5 mM EDTA buffer solution and vortexed. The contents were allowed to settle at 5°C, and the overlying liquid was decanted into a sterile tube. This step was repeated if further settlement of large particles was observed. The resulting liquids were spun at 10,000×g for 5 minutes, resulting in a pellet primarily composed of *Symbiodinium* cells and debris. This was followed by two spins at lower speed (500×g) to rinse the pellet and reduce host-derived material. While pellets from older specimens were visually similar to modern specimens in yield and color, microscopy revealed that older samples contained few intact theca, within which pigments were clearly degraded or absent.

### DNA Extraction, Quantification, and Amplification

Genomic DNA was extracted from whole *Symbiodinium* pellets using a Mo Bio Power Soil DNA isolation kit (Mo Bio Laboratories, Inc. Carlsbad, CA, USA). Pellets were first resuspended in buffer, transferred to bead tubes and spun at 10,000×g following the wet soil sample protocol. The resulting extracts were screened using ethidium bromide gel electrophoresis, and DNA concentrations and estimates of purity were determined using a NanoDrop spectrophotometer. Amplification of the ITS2 sub-region was conducted using the primers ‘ITSintfor2’ (forward) and ‘ITS2CLAMP’ (reverse) following the ‘touchdown’ amplification protocol described in Lajeunesse *et al.* (2002) using a BIO-RAD T100 thermal cycler. Post-PCR screening for bands of ∼300 bp revealed amplification success in 63 of 89 museum samples (70.7%). Troubleshooting on failed PCR reactions using different PCR recipes and various DNA polymerases was rarely successful, thus we attributed a failed PCR reaction to low DNA concentrations and/or DNA fragmentation. All amplicons were cleaned using an Exo:SAP enzyme protocol. Following cleanup, 1 µL of the PCR product was cycle sequenced in both directions using Big Dye 3.1 (Applied Biosystems, Foster City, USA). The resulting product was filtered through a Sephadex column, dried at 95°C, and directly sequenced using a 3730×l DNA analyzer (Applied Biosystems/Hitachi).

### Verification

A subset of museum specimens representing each host species (*n = *14), and the additional species *Eunicea succinea* (*n = *1) and *Pterogorgia anceps* (*n* = 1) were resampled to verify that our results could be repeated. All sample preparation, DNA extraction, PCR, and pre-sequencing reactions were conducted at a third, independent laboratory with no history of *Symbiodinium* research (EG3, NMNH). BLAST searches using these sequences aligned with *Symbiodinum* clades which corroborated our main conclusions. These data were included in the final analysis.

### Sequence Analysis

Chromatograms were screened for quality, edited, and complementary sequences were aligned using Geneious Pro v.5.4.6 (Biomatters Ltd.) [34]. Consensus sequences representing replicate samples of each species from each region/time were assembled and BLAST searches yielded close matching with *Symbiodinium* clade B. Thus, unique sequence characters from a minority of individuals (24%) were ignored by our analysis if they differed from the consensus sequence. We then aligned our sequences with known sequences of *Symbiodinium* sub-types within clade B obtained from GeoSymbio [32]. A phylogenetic tree was created with a clade A sequence (A3; Geosymbio) was used as an outgroup using neighbor-joining with 1,000 bootstrapped iterations. All sequences resulting from this study are archived in NCBI’s GenBank with the following accession numbers: KC461830-KC461901. URL: http://www.ncbi.nlm.nih.gov/genbank/.

## Results and Discussion

We have demonstrated that short genetic markers like the ITS2 sub-region of the nuclear rDNA are recoverable from *Symbiodinium* obtained from 6 species of dried gorgonian octocorals collected more than a century ago. Preliminary attempts at amplifying other common markers for genotyping, such as 18 s, were unsuccessful. Based on electrophoresis of genomic DNA extracts we observed high fragmentation, thus, given the relatively large size of 18 s (∼1800 bp) the probability of extracting a complete template was likely very low. However, amplification and sequencing of the smaller *Symbiodinium* ITS2 (∼200 bp) from dry-preserved living and historical octocorals specimens was successful and there was no apparent effect of preservation time on the percentage of samples that were successfully sequenced and aligned. Of the living specimens extracted, 52.2% resulted in high quality and aligned sequences compared to 65.8% of the museum specimens. This difference is probably due to experimental error in the process of extraction through sequencing and not a function of sample preservation. Indeed, DNA concentrations of modern sample extracts were as high as 38.6 ng µL^−1^ vs. 17.3 for museum specimens. However the mean DNA concentration obtained from living samples was no different than museum specimens (8.1 vs. 8.9 ng µL^−1^, Student’s t-test (two-tailed); *t = *0.33, df = 42, *p* = 0.74). These findings illustrate that sufficient *Symbiodinium* aDNA for genotyping can be obtained from small quantities of dry-preserved coral tissues, and suggests that drying is a tractable option for DNA preservation when the use of liquid fixatives is not feasible.

Our sequence data show that *Symbiodinium* types hosted by Caribbean octocorals collected between 98–172 years ago are indistinguishable from recently collected specimens. Both groups were found to contain representatives of clade B (Fig. 1). While our recent sample size was small, the results we obtained are in line with several previous works that have genotyped *Symbiodinium* from these host species [14], [18], [22], [32], [35]. Both recent and historical specimens of *G. ventalina,* and historical specimens of *G. flabellum,* and *P. acerosa* were 100% identical to GenBank sequences of *Symbiodinium* clade B1. *E. flexuosa* and *E. succinea* were found to host a different genotype, B1L, characterized by a substitution of cytosine for thymine at base pair 117, relative to B1 (Fig. 1). B1L has been previously described in symbiosis with *Eunicea spp.* [35] and other sea rods of the genus *Pseudoplexaura* and *Plexaurella* [36]. However, unique sequences that differed from the clade reference were found in 24% of the individuals. *E. flexuosa* had the highest occurrence of such characters with 4 out of 20 (5%) having 4 or fewer unique substitutions. One of these substitutions, a guanine for adenine substitution at base pair 189, was present in all four individuals which were all museum specimens collected at or before 1905. We found no similar SNP in GenBank or the Geosymbio database. While the sample size is low, we suggest that further sampling be conducted to determine if this is a new and/or extirpated *Symbiodinium* clade.

**Figure 1:**
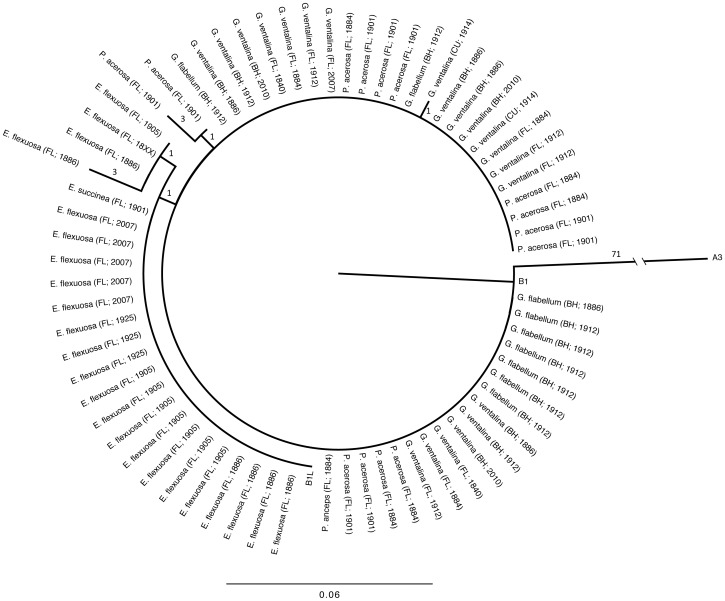
Phylogenetic tree of *Symbiodinium* extracted from various gorgonian host species based on ITS2 sequences from modern and museum-held specimens collected from Florida (FL), Cuba (CU), and the Bahamas (BH). Year of collection is noted in each branch label. These groups are shown in comparison to reference sequences from GeoSymbio from common clade B sub-types as well as a clade A3 sequence as an outgroup. Tree construction was based on neighbor-joining. Branch labels indicate the number of unique substitutions per sequence and branches are scaled to average substitutions per site, with the exception of A3 which was cropped.

Overall, there was no apparent genetic structure or clade-specificity based on the geographic location of collection. Specimens collected from Florida and the Bahamas consistently grouped together (Fig. 1). This is not surprising given that the majority of Caribbean gorgonians associate with clade B *Symbiodinium*. However, using microsatellite markers, Andras *et al.* (2011) recently illustrated significant genetic structure among clade B1 symbionts hosted by *G. ventalina* from the Bahamas and the Florida Keys, possibly due to the strong Florida Current preventing mixing among these populations [37]. Moreover, Finney et al (2010) used a combined ITS2/microsatellite approach to reveal that diversity within clade B is high, reflecting divergence among lineages that are host- and habitat-specific [36]. Thus, a logical future step is to target microsatellite loci of *Symbiodinium* B1 obtained from museum specimens for comparison with modern populations as such markers may be better suited for quantifying change in symbiont populations and testing the hypothesis that population level shifts in genetic diversity have occurred in response to global change [37], [38].

Our results yielded no evidence that there have been shifts in symbiont type hosted by gorgonian corals since human-induced global change. This finding poses two important questions; 1) is the gorgonian-algal symbiosis static and inflexible, and 2) has the severity of global change not been sufficient to drive major shifts in symbiont types hosted by gorgonian corals?

First, there is evidence that some gorgonians possess flexible symbioses as has been observed in several species of hard corals [39]. Newly settled polyps are capable of acquiring multiple symbiont types during early ontogeny [30]. As adults, several species have been found in symbiosis with clade A and C *Symbiodinium* as well as clade B [14], [19] and early studies may have failed to describe the presence of cryptic clades [35]. Yet, these examples of flexibility are apparently rare. More common are examples of symbiont stability over space and time. Goulet and Coffroth (2003) monitored *Symbiodinium* within individual colonies of *Plexaura kuna* and saw no clade-level variation over a period of 10 years [40]. Similarly, LaJeunesse *et al.* (2004) revealed that symbiont identities among an introduced population of *Fungia* retained their Pacific *Symbiodinium* 35 years after introduction to the Caribbean [41]. These examples are supportive of the hypothesis that host-symbiont associations are highly specific, reflecting a long evolutionary history [42].

Yet, it remains to be tested whether or not different symbiont types confer thermal tolerance to gorgonian hosts. This is an interesting hypothesis to test as bleaching is not uncommon among gorgonians, and the severity of bleaching appears to be species-specific [20]. For example, long-term records of temperature and symbiont densities of the sea rod *Plexaura kuna* from the Bahamas suggested that this species resists bleaching whereas other sea rods like *Plexaurella spp.* appear to bleach readily during warm periods [20], [23]. The potential for certain sub-clade types to enhance tolerance to environmental stress has recently been illustrated in hard corals [43]. Although differential bleaching susceptibility among Pacific Alcyonaceans is apparently not explained by *Symbiodinium* identity, this has yet to be tested in gorgonian octocorals [44].

Second, the bleaching threshold for many coral species is near maximal summer temperatures, therefore apparently small increases in ocean temperature have large consequences for increasing mass coral bleaching events. Coincident with these events are observations of differential mortality [45] and to a lesser extent symbiont shuffling among scleractinian coral species, primarily to *Symbiodinum* types which have been found to be tolerant to high irradiance (*e.g.* A3) or sedimentation and thermal stress (*e.g.* clade D) [46,47]. If we assume that zooxanthellate octocoral symbioses are functionally analogous to scleractinians, we might expect to find similar shifts among gorgonian corals over time. The absence of genetic evidence in this study may indicate that gorgonians are inflexible with respect to their symbiotic partners as may be the case for most corals [48], or perhaps gorgonians are overall more resilient in the midst of ocean warming. We contend that neither hypothesis is parsimonious and posit that significant changes in symbiont genotypes among coral host populations are not likely to be ecologically significant under the punctuated stress of climate change over the last century. This reflects the evolution of the symbiosis between Caribbean gorgonians and clade B *Symbiodinium* since the Pleistocene [42].

Our successful demonstration of aDNA extraction and amplification of informative taxonomic markers from *Symbiodinium* holds great promise for future studies. It has been argued that scleractinian corals are more flexible in their symbiotic associations than gorgonians, particularly at the clade level [16], [17]. If this is true, aDNA studies of scleractinian corals is a high priority for future research, though a significant challenge exists in finding intact tissues or skeletal reservoirs of preserved *Symbiodinium* cells. We attempted DNA extraction from one museum specimen of *Montastrea cavernosa* (NMNH #255089), collected in 1864, which appeared to have some remaining surface tissues. Unfortunately, we recovered very small quantities of DNA and were unable to amplify ITS2. Even so, milling of subsurface skeletal materials from archived scleractinians, and perhaps even fossil and sub-fossil skeletons may yet yield preserved *Symbiodinium* containing sufficient DNA for genotyping and warrants further study.
